# Supplementary material for: Magnetically Activated Piezoelectric 3D Platform Based on Poly(Vinylidene) Fluoride Microspheres for Osteogenic Differentiation of Mesenchymal Stem Cells
Source: Gels. 2022 Oct 20;8(10):680. doi: 10.3390/gels8100680 (PMC9602007; doi:10.3390/gels8100680)
Supplement: Supplementary file 1 [file gels-08-00680-s001.zip › Supplementary_Information.pdf]

Supplementary Materials for:

Magnetically Activated Piezoelectric 3D Platform Based on Poly(Vinylidene) Fluoride Microspheres for Osteogenic Differentiation of Mesenchymal Stem Cells

Maria Guillot-Ferriols [1,2,*], María Inmaculada García-Briega [1,2], Laia Tolosa [2,3], Carlos M. Costa [4,5,6], Senentxu Lanceros-Méndez [4,5,7,8], José Luis Gómez Ribelles [1,2], Gloria Gallego Ferrer [1,2]

[1] Centre for Biomaterials and Tissue Engineering (CBIT), Universitat Politècnica de València, 46022 Valencia, Spain
[2] Biomedical Research Networking Center on Bioengineering, Biomaterials and Nanomedicine, Carlos III Health Institute (CIBER-BBN, ISCIII), 46022 Valencia, Spain
[3] Experimental Hepatology Unit, Health Research Institute La Fe (IIS La Fe), 46026 Valencia, Spain
[4] Physics Centre of Minho and Porto Universities (CF-UM-UP), University of Minho, 4710-057 Braga, Portugal
[5] Laboratory of Physics for Materials and Emergent Technologies, LapMET, University of Minho, 4710-057 Braga, Portugal
[6] Institute of Science and Innovation for Bio-Sustainability (IB-S), University of Minho, 4710-057 Braga, Portugal
[7] BCMaterials, Basque Center for Materials, Applications and Nanostructures, UPV/EHU Science Park, 48940 Leioa, Spain
[8] IKERBASQUE, Basque Foundation for Science, 48009 Bilbao, Spain.
* Correspondence: maguifer@upv.es (M.G.-F.)

Table S1. Primer sequences used for Real-time qPCR. Alkaline phosphatase (ALP), Collagen type I (COL I), Runt-related transcription factor 2 (RUNX2), Osteocalcin (OCN), glyceraldehyde-3-phosphate dehydrogenase (GAPDH).

Gene	Forward Primer (5′-3′)	Reverse Primer (5′-3′)	Annealing Temperature (°C)
ALP	ATGAAGGAAAAGCCAAGCAG	CCACCAAATGTGAAGACGTG	55.2
COL I	GCCAAGACGAAGACATCCCA	GGCAGTTCTTGGTCTCGTCA	59.5
RUNX2	TCACAAATCCTCCCCAAGTA	GGCGGTCAGAGAACAAACTA	55.7
OCN	GTGCAGAGTCCAGCAAAGGT	TCAGCCAACTCGTCACAGTC	59.2
GAPDH	GTCTCCTCTGACTTCAACAGCG	ACCACCCTGTTGCTGTAGCCAA	62.4

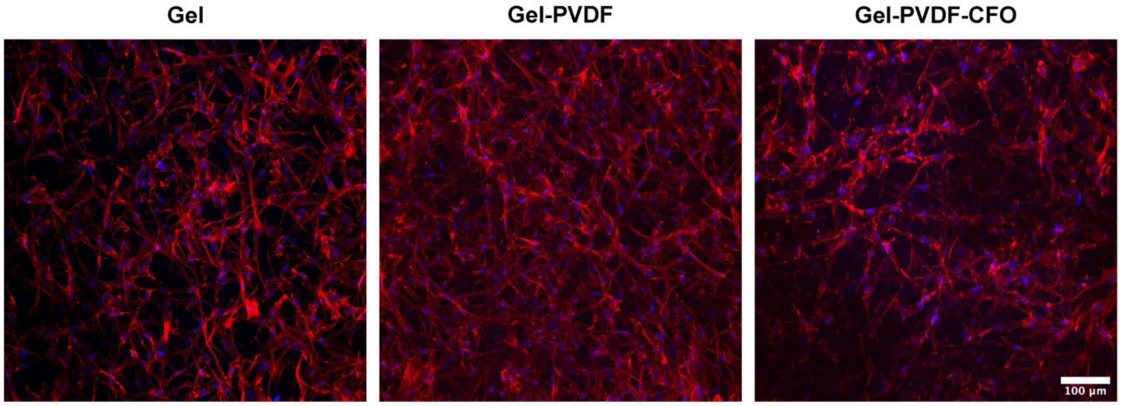

Figure S1. Representative Z projection of non-cryosectioned Gel, Gel-PVDF and Gel-PVDF-CFO hydrogels after 14 days in culture in static condition. Actin cytoskeleton appears in red and cell nuclei in blue. Scale bar 100 µm.
